# Applying CRISPR Technologies for the Treatment of Human Herpesvirus Infections: A Scoping Review

**DOI:** 10.3390/pathogens14070654

**Published:** 2025-07-01

**Authors:** Chloë Hanssens, Jolien Van Cleemput

**Affiliations:** Laboratory of Virology, Department of Translational Physiology, Infectiology and Public Health, Ghent University, Salisburylaan 133, 9820 Merelbeke, Belgium

**Keywords:** CRISPR-Cas, herpesvirus, antiviral therapy, gene therapy

## Abstract

Background: Human herpesviruses are double-stranded DNA viruses of which eight types have been identified at present. Herpesvirus infection comprises an active lytic phase and a lifelong latency phase with the possibility of reactivation. These infections are highly prevalent worldwide and can lead to a broad spectrum of clinical manifestations, ranging from mild symptoms to severe disease, particularly in immunocompromised individuals. Clustered regularly interspaced palindromic repeats (CRISPR)-based therapy is an interesting alternative to current antiviral drugs, which fail to cure latent infections and are increasingly challenged by viral resistance. Objective: This scoping review aimed to summarize the current state of CRISPR-based antiviral strategies against herpesvirus infections, highlighting the underlying mechanisms, study design and outcomes, and challenges for clinical implementation. Design: A literature search was conducted in the databases PubMed and Web of Science, using both a general and an individual approach for each herpesvirus. Results: This scoping review identified five main mechanisms of CRISPR-based antiviral therapy against herpesvirus infections in vitro and/or in vivo. First, CRISPR systems can inhibit the active lytic replication cycle upon targeting viral lytic genes or host genes. Second, CRISPR technologies can remove latent viral genomes from infected cells by targeting viral genes essential for latency maintenance or destabilizing the viral genome. Third, reactivation of multiple latent herpesvirus infections can be inhibited by CRISPR-Cas-mediated editing of lytic viral genes, preventing a flare-up of clinical symptoms and reducing the risk of viral transmission. Fourth, CRISPR systems can purposefully induce viral reactivation to enhance recognition by the host immune system or improve the efficacy of existing antiviral therapies. Fifth, CRISPR technology can be applied to develop or enhance the efficiency of cellular immunotherapy. Conclusions: Multiple studies demonstrate the potential of CRISPR-based antiviral strategies to target herpesvirus infections through various mechanisms in vitro and in vivo. However, aspects regarding the delivery and biosafety of CRISPR systems, along with the time window for treatment, require further investigation before broad clinical implementation can be realized.

## 1. Introduction

Eight human herpesviruses have been identified so far, including herpes simplex virus type 1 and 2 (HSV-1 and -2), varicella-zoster virus (VZV), Epstein-Barr virus (EBV), human cytomegalovirus (HCMV), human herpesvirus 6 and 7 (HHV-6 and -7), and Kaposi’s sarcoma-associated herpesvirus (KSHV) [1]. All herpesviruses are widespread, with (sero)prevalences varying by herpesvirus type and geographic region. It is highest for HSV-1, EBV, HCMV, HHV-6, and HHV-7, estimated to exceed 90% for some types [2,3,4,5,6,7,8]. Herpesviruses share a common virion structure composed of three major elements: (1) a nucleocapsid containing a large, linear double-stranded DNA genome encapsulated by an icosahedral capsid, (2) an envelope consisting of a host-derived lipid bilayer containing different viral glycoproteins, and (3) the tegument, a proteinaceous layer between the capsid and the envelope [9]. Another hallmark of herpesviruses is their biphasic life cycle, consisting of lytic and latent phases. The lytic replication cycle results in the production of virus particles and is orchestrated through a cascade of gene transcription. First, the expression of immediate early (IE) genes is initiated, which subsequently drives the transcription of early (E) genes involved in viral genome replication. Following DNA replication, late (L) genes are transcribed into proteins involved in virus assembly [9]. Following the lytic phase, herpesviruses establish a lifelong latent infection within host cells, either as episomal DNA or, in some cases, integrated into the host genome in a latent state [10]. The latent phase is characterized by limited viral gene expression and the absence of virion production [9,10]. Reactivation of latent virus can be triggered by various stimuli such as ultraviolet light, physical or emotional stress, and fever, leading to the reinitiation of the lytic replication cycle and production of new virions [9,11].

Herpesviruses infect a broad range of cells and tissues, depending on the virus and host context. Clinical manifestations vary widely and are associated with specific tissues involved during lytic replication. Infected individuals may remain asymptomatic or present with mild symptoms such as fever, malaise, and lymphadenopathy [12,13,14,15,16,17,18,19,20,21]. Dermatological manifestations are also common. However, in immunocompromised patients, herpesvirus infection can lead to severe complications, such as encephalitis, meningitis, hepatitis, pneumonia, and various neuropathies [12,17,20,22,23,24,25]. Notably, two herpesviruses, EBV and KSHV, are oncogenic and have been associated with the development of malignancies such as lymphomas, nasopharyngeal, gastric, and smooth muscle carcinomas and sarcomas, following the transformation of latently infected cells [18,19,26,27].

Current herpesvirus infection treatment options rely predominantly on antiviral drugs such as nucleoside-, nucleotide-, and pyrophosphate analogs, which inhibit the viral DNA polymerase and therefore virus replication and virion production [28]. Given that the viral DNA polymerase is active only during lytic replication, these antiviral drugs are only able to treat patients symptomatically without eliminating the latent virus [28]. Furthermore, multiple herpesviruses have already evolved resistance mechanisms to circumvent the action of these drugs [1].

In order to cure both active and latent herpesvirus infections, herpesviral DNA should be eliminated from infected cells. Genetic therapy would be an elegant approach to reach this goal. The discovery and subsequent development of a very precise, relatively simple, and versatile genome-editing tool known as CRISPR-Cas has boosted genetic therapy technologies over the recent years. The CRISPR-Cas9 system, short for clustered regularly interspaced short palindromic repeats and CRISPR-associated protein 9, was originally derived from a bacterial adaptive immune mechanism [29]. It functions by guiding the endonuclease Cas9 to specific DNA sequences using a guide RNA (gRNA), resulting in targeted double-stranded breaks. These breaks are then repaired by either non-homologous end joining (NHEJ) or homology-directed repair (HDR) if a repair template is available [30,31,32]. NHEJ is faster and more efficient than HDR, but is inherently error-prone, often resulting in insertions or deletions (INDELs) at the cut site. INDEL formation in a gene of interest can result in loss of function, frameshift mutations, or the creation of a premature stop codon, leading to gene knock-out (KO). As an antiviral strategy, this approach is particularly useful for targeting essential viral genes, resulting in replication-defective viruses and ultimately the clearance of infection. However, there is a risk of mutational escape, whereby the virus accumulates sequence changes that prevent recognition by the original gRNA while retaining partial or full functionality. To mitigate this, multiple gRNAs can be introduced to target several sites within the viral genome, thereby increasing the frequency of double-strand breaks. This multiplexing strategy can lead to large genomic deletions and loss of viral genome stability, ultimately resulting in complete viral genome clearance. However, when applied to integrated viral genomes, such extensive genome editing may also pose a risk of chromosomal instability or large-scale host genomic rearrangements. The HDR pathway is more accurate, but less efficient than NHEJ. In the context of viral genome engineering, HDR is primarily employed for knock-in applications, such as the insertion of transgenes to generate viral vectors or gene drive viruses.

Besides the CRISPR-Cas9 system, other CRISPR systems with different Cas enzymes have been developed, such as CRISPR-CasX, which also applies specific double-stranded breaks using the smaller endonuclease CasX [33]. Two different CRISPR systems, namely CRISPR interference (CRISPRi) and CRISPR activation (CRISPRa), do not create double-stranded breaks but transiently modulate transcription by using an endonucleolytically deactivated Cas9 (dCas9) protein [34]. More specifically, the dCas9 protein can be fused to transcriptional repressor or activator domains to temporarily inhibit or activate gene expression, enabling CRISPRi or CRISPRa, respectively [34].

Given its sequence-specific DNA-targeting ability, CRISPR-Cas represents an attractive approach for antiviral therapy, particularly for targeting and disrupting herpesviral genomes. The application of the CRISPR-Cas editing tool has already been explored in many fields, including biotechnology and medicine, targeting genomes in both prokaryotes and eukaryotes. Applications include disease modeling to investigate pathogenesis/oncogenesis, genome-wide loss-of-function screening, and gene therapy for genetic disorders [31]. This scoping review aimed to investigate the application of CRISPR-Cas systems as antiviral therapy against human herpesviruses. We summarize findings from 43 primary studies to provide an overview of the current state of research and mechanisms by which CRISPR systems are being applied to treat herpesvirus infections.

## 2. Methods

### 2.1. Search Strategy, Eligibility Criteria, and Selection Process

The search strategy and eligibility criteria of this scoping review were drafted and agreed upon by both authors using the Preferred Reporting Items for Systematic Reviews and Meta-Analyses extension for Scoping Reviews (PRISMA-ScR) [35]. The protocol for this scoping review has been registered in the Open Science Framework (OSF) Registries database and is publicly accessible at https://doi.org/10.17605/OSF.IO/K4BTW (accessed on 5 June 2025). We searched the databases PubMed and Web of Science using free text and/or medical subject headings (MeSH) terms, as detailed in Table 1. The search covered all records written in English up to 16 March 2025. In addition, the citation searching method was also used to identify one additional article. All identified publications were pooled in EndNote 21 (Clarivate Analytics, Philadelphia, PA, USA) prior to duplicate removal. Subsequently, an initial screening of the articles was conducted based on the relevance of their titles and abstracts and full-text availability. Finally, after full-text reading of the remaining articles, publications with the following criteria were excluded: (1) contextual irrelevance to the subject of this review (absence of CRISPR application, absence of application to human herpesviruses, or absence of therapeutical strategy), (2) type of article (review or meeting abstract), and (3) absence of result descriptions.

### 2.2. Data Charting Process and Synthesis of Results

Data on the design and results of studies using CRISPR technology as antiviral therapy against herpesviruses were extracted. Specifically, for the design of the studies, data regarding the test subject, the CRISPR system, and the delivery vector were abstracted. The data were first synthesized according to the targeted herpesvirus type, followed by the targeted genes, and the chosen mechanism of CRISPR-mediated antiviral therapy. Individual characteristics such as the test subject, the CRISPR system, and the delivery vector are described for each study separately. A summary table (Table 2) is included to provide a schematic overview of the main design characteristics of the experiments conducted in the included studies.

## 3. Results

### 3.1. Screening Results

**Figure 1:**
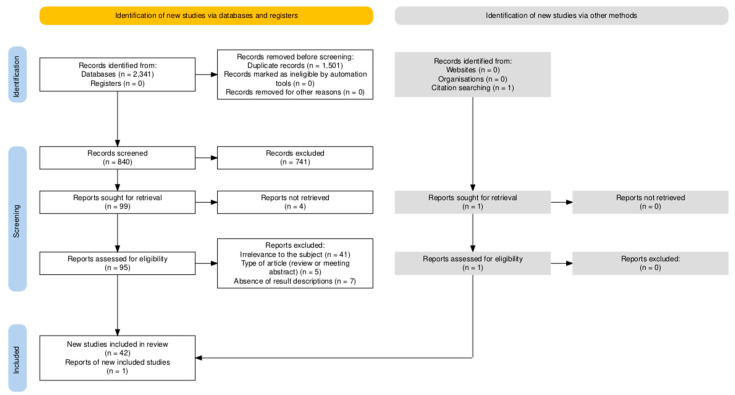
PRISMA Flow Diagram, generated with the PRISMA Flow Diagram tool [36], schematically visualizing the article selection process.

A total set of 2341 identified publications was pooled in EndNote 21, where duplicate removal reduced the set to 840 unique records. Subsequently, an initial screening of the articles was conducted based on the relevance of their titles and abstracts, resulting in the exclusion of 741 articles. Additionally, four articles were excluded based on full-text unavailability. Finally, a full-text reading of the remaining 95 articles resulted in the inclusion of 43 articles after 52 publications were excluded based on the following criteria: (1) contextual irrelevance to the subject of this review (absence of CRISPR application, absence of application to human herpesviruses, or absence of therapeutical strategy), (2) type of article (review or meeting abstract), and (3) absence of result descriptions. Figure 1 displays the PRISMA flow diagram, generated with the PRISMA Flow Diagram tool [36], which provides a schematic visualization of the article selection process.

### 3.2. Overview of CRISPR Strategies Used to Target Different Human Herpesviruses

**Figure 2:**
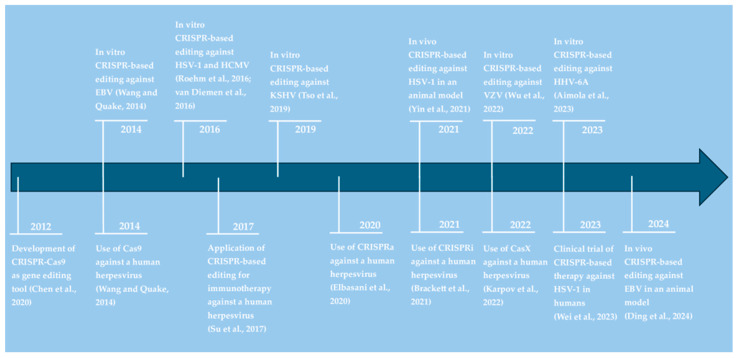
Timeline visualizing the application of CRISPR technologies against herpesvirus infections. References in alphabetical order: Aimola et al., 2023 [37]; Brackett et al., 2021 [38]; Chen et al., 2020 [30]; Ding et al., 2024 [39]; Elbasani et al., 2020 [40]; Karpov et al., 2022 [41]; Roehm et al., 2016 [42]; Su et al., 2017 [43]; Tso et al., 2019 [44]; van Diemen et al., 2016 [45]; Wang and Quake, 2014 [46]; Wei et al., 2023 [47]; Wu et al., 2022 [48]; Yin et al., 2021 [49].

Table 2, included at the end of the results section, provides an overview of CRISPR-based antiviral strategies against human herpesviruses and the respective targeted genes. As illustrated in Figure 2, it took only 2 years from the initial demonstration of CRISPR-Cas9 as a gene-editing tool in 2012 to its application as an antiherpesviral strategy. Over the following decade, different CRISPR systems have been developed and tested in vitro and in vivo to target six out of eight known human herpesviruses (Figure 2). These approaches are discussed in more detail below for each virus.

#### 3.2.1. CRISPR Systems Targeting Herpes Simplex Virus Type 1

##### The Application of CRISPR Systems to Inhibit Lytic HSV-1 Replication

Several studies [41,42,45,50,51,52,53,54,55,56,57,58,59,60,61] used the CRISPR system to edit HSV-1 genes to interfere with lytic virus replication. First, IE genes represent an interesting target as they play an essential role in initiating the full replication cycle. Three studies [41,42,52] targeted the *infected cell protein 0* (*ICP0*) gene using a CRISPR-Cas9 system. Transfection of a monkey cell line, Vero L7 cells, with anti-*ICP0* gRNA- and CRISPR-Cas9-carrying plasmids compromised HSV-1 replication and inhibited ICP0-mediated disruption of host antiviral promyelocytic leukemia (PML) nuclear bodies [42]. Similarly, lentiviral transduction of another HSV-1-infected Vero cell line with anti-*ICP0* gRNAs and CRISPR-Cas9 reduced viral titers in another study [52]. However, a third study [41] could not confirm that targeting *ICP0* expression in Vero cells inhibits HSV-1 replication or infectivity, but this could be related to differences in multiplicity of infection (MOI) between the three studies [41,42,52]. Transducing human oligodendroglioma Tübingen (TC620) cells with Cas9 plus anti-*ICP0* gRNAs abrogated the full HSV-1 replication cycle [42]. Indeed, the production of IE and subsequently E and L genes, as well as virus particle production, was reduced [42]. Interestingly, uninfected cells expressing Cas9 and anti-*ICP0* gRNAs were almost completely protected from HSV-1 infection [42]. Targeting a different IE gene, namely *ICP4*, by CRISPR-Cas9 via lentiviral transduction also resulted in almost complete inhibition of HSV-1 infection in Vero cells and reduced viral titers in human foreskin fibroblasts [52,56]. Additionally, transducing murine primary trigeminal ganglion neuronal cultures, which present a representative in vitro model for herpesvirus latency sites, with an adeno-associated virus (AAV) serotype 1 (AAV1) encoding Cas9 and a gRNA targeting *ICP4* inhibited HSV-1 replication, as evidenced by a marked reduction in HSV-1 genome copies [52]. Targeting the *ICP27* gene in Vero cells and human foreskin fibroblasts through lentiviral delivery of a CRISPR-Cas9 system also impaired HSV-1 replication [45,56]. Lastly, three studies [42,50,51] applied a multiplex strategy, simultaneously targeting two sites of the same IE gene or two separate IE genes. Simultaneous transfection of Vero cells with two plasmids carrying Cas9 and gRNAs against different sites of *ICP0* or *ICP27*, followed by HSV-1 infection, significantly reduced HSV-1 loads and titers [50]. This was further enhanced upon AAV2-mediated delivery of the CRISPR systems [50,51]. Similar results were observed in a different study [42], as simultaneously targeting two genes, namely *ICP0*-*ICP4*, *ICP0*-*ICP27*, or *ICP4*-*ICP27*, in TC620 cells via lentiviral transduction of a CRISPR-Cas9 system resulted in the complete absence of HSV-1 infection.

HSV-1 E genes are involved in viral DNA replication and have been targeted by multiple studies [41,42,45,53,54,57,58]. Delivery of a CRISPR-Cas9 system individually targeting the *unique long 5* (*UL5*), *UL8*, *UL9*, *UL19*, *UL29*, *UL42*, or *UL52* gene via lentiviral vectors or plasmid transfection into Vero cells and/or human foreskin fibroblasts significantly impaired HSV-1 replication in multiple studies [41,45,54,56]. An alternative method for CRISPR-Cas delivery, namely engineered extracellular vesicles (EVs), was applied in a different study [58]. Delivery of engineered EVs carrying Cas9 and a gRNA against *UL29*, encoding ICP8, in HSV-1-infected Vero cells and/or Hela cells decreased *glycoprotein D (gD)* and *viral protein 16* (*VP16*) transcription, ICP8 and gD production, and the viral plaque number. Labeling the EVs with a neuro-targeting rabies virus glycoprotein peptide (RVG29) increased the targeting-specificity of the EVs toward nervous tissues both in vitro and in vivo. RVG-labeled EVs were even able to transverse endothelial cells to invade underlying neuronal hippocampal terminal (HT22) cells in vitro, indicating they could penetrate blood vessels. Administering the RVG-Cas9-UL29-gRNA EVs in vivo before HSV-1 infection significantly reduced the viral titer in the serum and resulted in less severe clinical symptoms and higher survival rates [58]. Four studies [41,45,54,56] used a CRISPR-Cas9 system to target *UL30*, resulting in reduced HSV-1 replication in Vero cells and human foreskin fibroblasts. Besides INDELs in the HSV-1 genome, linear DNA species were also identified, indicating that CRISPR-Cas9 additionally inhibits HSV-1 by inducing large genome deletions and DNA degradation [56]. Besides CRISPR-Cas9, CRISPR-CasX targeting *UL30* was also able to reduce HSV-1 infection in Vero cells [41]. A different E gene, namely *UL39*, encoding the ICP6 protein involved in viral replication, was targeted using the CRISPR-Cas9 system in two different studies [53,57]. Delivery of Cas9 and anti-*UL39* gRNAs substantially reduced viral loads in vitro in HSV-1-infected Vero cells following plasmid transfection and in vivo in brain tissues of HSV-1-infected Bagg albino laboratory-bred substrain c (BALB/c) mice upon topical eye application of the CRISPR therapy [53,57]. Three studies [41,45,54] used a multiplex strategy to simultaneously target two E genes and observed higher antiviral effects compared to a singleplex strategy. More precisely, lentiviral transduction or plasmid transfection of Vero cells with a CRISPR-Cas9 system simultaneously targeting *UL8*-*UL29*, *UL8*-*UL52*, *UL29*-*UL52*, or *UL19*-*UL30* inhibited viral replication, even reaching an HSV-1-free Vero cell population in one study [41,45,54]. Additionally, double gene-targeting of *UL8*-*UL29* or *UL29*-*UL52* in HSV-1-infected Medical Research Council strain 5 (MRC5) cells via lentiviral delivery of a CRISPR-Cas9 system resulted in the complete loss of infectious particles [45].

In line with the IE and E genes, targeting the HSV-1 L genes impaired virus replication. For instance, targeting the L HSV-1 *gD* gene, encoding the gD envelope glycoprotein, using a CRISPR-gRNA-Cas9 plasmid significantly reduced HSV-1 titers in human embryonic kidney (HEK293)-AD cells [55]. Additionally, lentiviral transduction of Vero cells with a CRISPR-Cas9 system targeting *UL15*, *UL27*, *UL36*, or *UL37* resulted in impaired HSV-1 replication [45].

Interestingly, even CRISPR-Cas9-mediated knock-out of nonessential genes (*unique short 3* [*US3*] or *US8*) or non-coding regions (intergenic regions between *UL26*-*UL27* and *UL37*-*UL38*) was able to reduce HSV-1 replication in Vero cells and human foreskin fibroblasts, respectively [45,56]. This reduction is likely due to defects in virus particle assembly arising from unrepaired dsDNA breaks, as the rapid HSV-1 replication kinetics exceed cellular DNA repair capacities, resulting in impaired infection of naïve cells [45].

Instead of viral genes, one study [61] targeted the host gene *double homeobox 4* (*DUX4*), of which the translated protein is involved in the transcription activation of HSV-1 E and L genes upon stimulation by HSV-1 IE proteins. Indeed, lentiviral transduction of a haploid human (HAP1) cell line and HEK 293T cells with a CRISPR-Cas9 system against *DUX4*, followed by HSV-1 infection, impaired HSV-1 replication. As it was observed that all human herpesviruses induce the expression of DUX4, DUX4 knock-out could potentially impair the replication of other herpesviruses as well [61].

Lastly, Ying et al. [60] took an approach of multi-point genome editing by designing a strategy named coordinated lifecycle elimination against viral replication (CLEAR). CLEAR is based on the simultaneous editing of IE (*ICP4* and *ICP27*) and L (*VP16*, *gD*) genes, both active during different steps in the virus life cycle. Targeting each gene individually upon plasmid delivery of the CRISPR system to baby hamster kidney (BHK)-21 cells, followed by HSV-1 infection, significantly reduced the viral titer, noting the highest and lowest efficiency upon targeting *ICP4* and *VP16*, respectively. This antiviral effect was doubled when all genes were targeted simultaneously in the CLEAR strategy. In case BHK-21 cells were first infected with HSV-1 prior to CLEAR treatment, virus inhibition was the most prominent in the early phase post-transfection, suggesting a small time window of less than 12 h for treatment. In vivo, intracerebral injection of lentiviruses carrying the CRISPR-Cas9 system in murine prevention models reduced viral replication at the injection site and prevented viral transmission to downstream brain areas. These antiviral effects were more prominent with the CLEAR strategy compared to individual *ICP4*- *or ICP27*-targeting. In line with the prevention model, HSV-1 proliferation and transmission were significantly inhibited in murine treatment models, receiving the treatment 1 day post-HSV-1 infection [60].

Simultaneous editing of one or more E and L genes was also achieved using a unique delivery mechanism. More precisely, Wang et al. [59] designed autonomous, intelligent, virus-inducible immune-like (ALICE) systems that can autonomously detect intracellular pathogens and subsequently activate antiviral responses. The development of ALICE cells is based on generating transgenic HEK293 cells with SV40 large T antigen (HEK293T cells), stably expressing a destabilized STING (stimulator of IFN genes) protein in the cytoplasm which detects intracellular exogenous dsDNA or RNA and subsequently activates a synthetic immune signaling pathway, resulting in the expression of a given gene of interest under the control of a virus-inducible promoter P_ALICEX_. Two interesting ALICE systems for treating HSV-1 infection include ALICE_Cas9_ and ALICE_Ab_, as they result in Cas9 and HSV-1-specific gRNA expression and the production of HSV-1 monoclonal neutralizing antibodies E317, respectively, upon detection of HSV-1 DNA. Both systems can also be combined (ALICE_Cas9+Ab_). In vitro, all three systems, but mostly the ALICE_Cas9+Ab_ system, inhibited HSV-1 replication in and transmission among the HEK293T cells. In vivo, prevention, but also treatment mouse models showed reduced HSV-1 mRNA expression and virus titer in different tissues following intraperitoneal implantation of hydrogels containing transgenic HEK_ALICE-SEAP-Cas9_ (ALICE_Cas9_ with gRNAs targeting the HSV-1 genes *US8* [L], *UL29* [E], or *UL52* [E]), transgenic HEK_ALICE-E317Ab_ (ALICE_Ab_), and/or transgenic HEK_ALICE-Cas9-E317Ab_ (ALICE_Cas9+Ab_) cells [59].

##### The Application of CRISPR Systems to Edit Quiescent HSV-1 Genomes and Disrupt Viral Reactivation

Transducing an HSV-1 latency model of human cerebral organoids with an AAV2 vector carrying Cas9 and two gRNAs against the IE *ICP0* or *ICP27* gene reduced HSV-1 reactivation rates by 2.5 to 5-fold, respectively, compared to control upon the induction of reactivation [51]. Two studies [45,56] applied the CRISPR-Cas9 system to quiescently HSV-1-infected MRC5 human lung or foreskin fibroblasts by lentiviral transduction of Cas9 and gRNAs targeting the viral E genes *UL8*, *UL29*, *UL30*, or *UL52*. In both cell types, all single gRNA-Cas9 complexes resulted in a reduction in viral reactivation upon stimulation [45,56]. This effect was enhanced following the simultaneous delivery of two gRNA-Cas9 complexes targeting *UL29* and *UL30* [56]. However, in both studies, no loss of HSV-1 genomes nor the induction of large INDELs was detected, suggesting that the CRISPR-Cas9 system had low efficiency in editing quiescent HSV-1 genomes compared to lytic viral genomes [45,56].

##### The Application of CRISPR Systems for the Prevention or Treatment of Herpes Simplex Keratitis

As HSV-1 is the leading infectious cause of blindness, multiple studies [47,49,50,59,62] have explored the application of CRISPR-Cas systems for herpes simplex keratitis (HSK) therapy. A first approach implied in vivo corneal scarification in HSV-1 latently infected rabbit keratitis models to deliver CRISPR-Cas9-carrying AAVs (type 8 or 9) targeting *ICP0* and *ICP27*, which resulted in absent viral shedding in the tear swabs of approximately 50–60% of the treated eyes upon virus reactivation [50]. Still, AAV presence in the cornea and the trigeminal ganglia was low. Higher vector concentrations could be achieved following intravenous injection of the CRISPR-Cas9 AAVs. All eyes with local AAV presence showed a complete absence of viral shedding. Additionally, a 50% reduction of HSV-1 viral load and up to 80% reduced transcription of the latently expressed *latency-associated transcript* (*LAT*) gene were observed in the trigeminal ganglia, indicating partial HSV-1 removal from the latent reservoir [50].

A second approach explored the application of ALICE systems in an established HSK mouse model [59]. Two AAVs encoding ALICE_Cas9_ and gRNAs targeting *ICP4* or ALICE_Ab_ were administered intravenously prior to HSV-1 infection. This led to significantly reduced viral titers in the trigeminal ganglia, eye, and brain samples as well as a complete blockage of HSV-1-induced upregulation of different inflammatory molecules [59].

A third approach involved the development of HSV-1-erasing lentiviral particles (HELP) carrying Cas9 mRNA for transient Cas9 expression and two gRNAs simultaneously targeting the HSV-1 genes *UL8* and *UL29* [47,49]. In vitro, HELP transduction of HEK293T cells, spontaneously immortalized human keratinocyte (HaCaT) cells, and murine primary corneal stromal cells, as well as in tissue cultures of human corneas, followed by HSV-1 infection, partially inhibited HSV-1 production. Next, in vivo experiments were conducted on murine HSK models via intrastromal injection of HELP into the corneas. Interestingly, HELP was detected in trigeminal ganglia, indicating that these vectors can be retrogradely transported along axons from neurons innervating the cornea. A treatment prevention model showed that HELP injection prior to HSV-1 infection was able to reduce viral loads in eye, trigeminal ganglion, and brain samples and thus, inhibit viral transmission. Consequently, HELP treatment inhibited disease progression, corneal expression of inflammatory molecules, and infiltration of inflammatory cells. Also, in a therapeutic murine model, in which mice received HELP treatment after HSV-1 infection, HSV-1 replication in the corneas and trigeminal ganglia, as well as lesion formation in the eyelids, and disease progression were diminished. Additionally, HELP treatment decreased HSV-1 loads in both the eyes and the trigeminal ganglia of HSV-1 latently infected mice, which received a reactivation stimulus prior to HELP administration [49]. Finally, HELP treatment was tested in a clinical trial in which three patients suffering from severe refractory HSK and acute corneal perforation were intrastromally injected with HELP during penetrating keratoplasty (NCT04560790) [47]. All examined tear swabs and corneal button samples remained negative for HSV-1 in the average 18-month follow-up, and no patient showed HSK recurrence. Adverse effects were primarily associated with the elevated risk of penetrating keratoplasty or the use of glucocorticoids post-surgery. Additionally, the HELP treatment seemed to be tolerated by the host immune system, as no vector-specific IgG antibodies were detected [47].

A fourth approach for potential HSK treatment implies targeting host gene expression instead of viral gene expression. Specifically, the *nectin cell adhesion molecule 1* (*NECTIN-1*) gene is an interesting target as it encodes a gD receptor on the cornea important for the invasion of the virus by interaction with its glycoprotein D [62]. Lentiviral transduction of human corneal epithelial cells with a CRISPR-Cas9 system targeting exon two of *NECTIN-1*, followed by HSV-1 infection, resulted in strongly decreased HSV-1 infection and HSV-1 DNA load [62].

#### 3.2.2. CRISPR Systems Targeting the Lytic Reproduction Cycle and Reactivation of Varicella-Zoster Virus

One study [48] observed partial inhibition of VZV lytic replication in adult retinal pigment epithelial clone 19 (ARPE-19) cells and human embryonic stem cell (hESC)-derived neurons following AAV2-mediated transduction of the CRISPR-Cas9 system targeting the duplicated IE *open reading frame (ORF)62/71* gene encoding the major viral transcription factor ICP4 homolog protein. Applying the same CRISPR strategy on latently infected hESC-derived neuron cultures similarly resulted in reduced VZV replication upon virus reactivation [48].

#### 3.2.3. CRISPR Systems Targeting Epstein-Barr Virus

##### The Application of CRISPR Systems to Edit EBV Latency-Associated Genes

Simultaneously targeting three latency-associated genes involved in maintaining viral episomes and/or host cell transformation (i.e., *EBV nuclear antigen 1* or *EBNA1*, *EBNA3C*, and *latent membrane protein 1* or *LMP1*) and three structural repeat regions (PstI repeats, EBNA-leader protein (LP) repeats, and 125bp repeats) in EBV-infected Raji cells with a mixture of seven gRNAs and Cas9, administered via plasmid transfection, resulted in a decrease in EBV load, reaching almost complete elimination in a quarter of the cell population. It also resulted in complete suppression of cell proliferation and a decrease in total cell count, indicating the initiation of cell apoptosis [46]. These findings suggest that CRISPR-Cas9 editing of the EBV genome can restore the apoptotic pathway previously interrupted by EBV [46]. While targeting only the repeat regions led to a complete suppression of cell proliferation, only a small decrease in EBV load was observed. Vice versa, targeting the EBNA1 coding region strongly reduced the viral load without completely suppressing cell proliferation [46]. This was confirmed by another study [63] using a different infection model. Targeting the *EBNA1* coding region, *latent origin of replication* (*OriP*), or W repeats individually with EBV-specific CRISPR-Cas9-encoding plasmids reduced virion production in HEK293 cells carrying the M81 EBV strain. In latently EBV-infected nasopharyngeal carcinoma (NPC) cells, viral DNA loads decreased progressively over a period of 4 weeks post-transfection with CRISPR-Cas9 plasmids targeting *EBNA1*, *OriP*, and/or W repeats. However, in all experiments, the CRISPR system failed to completely eradicate the EBV episomes or alter cell viability and proliferation, even when the dose was increased. Still, the NPC cells were sensitized to chemotherapeutic killing following CRISPR treatment [63]. Targeting *LMP1* or *LMP2A* by transfection of EBV-CRISPR-Cas9 plasmids in another type of EBV-infected NPC cells (Cantonese nasopharyngeal epithelial cells or CNE-2 cells) inhibited EBV replication [64]. Even cell growth was significantly reduced upon *LMP1* targeting [64]. Disruption of *LMP1* in a third type of EBV-infected NPC cell line (NPC C666-1) by another CRISPR-Cas9 delivery method (i.e., poly[β-amino ester] or PBAE-plasmid polyplex nanoparticles [NPs]) also reduced tumor cell viability, cell growth, and the ability to form colonies [65]. In vivo, the delivery of these CRISPR-Cas9 polyplex NPs in murine EBV-NPC xenograft tumor models significantly inhibited tumor growth and induced tumor cell apoptosis, with tumor inhibition rates up to 45–81% [39,65]. Finally, lentiviral transduction of Burkitt’s lymphoma Akata-Bx1 cells with a singleplex CRISPR-Cas9 system targeting *EBNA1* or *OriP* resulted in the loss of viral gene transcription and EBV genomes in approximately half the cells [45]. Sequential transduction with two lentiviral vectors carrying different gRNAs against *EBNA1* and/or *OriP* strongly enhanced these inhibitory effects on virus replication. The highest efficiency was noted upon double targeting of *EBNA1*, as more than 95% of the cells were cleared from the EBV genome [45].

##### The Application of CRISPR Systems to Induce the Reactivation of Latent EBV Infection

One study [66] applied CRISPRa to target the EBV IE gene *Z trans-activator (ZTA)* promoter region in EBV-positive B and epithelial cancer cells via lentiviral transduction to achieve EBV reactivation and consequently sensitize the cells to ganciclovir treatment. Almost all CRISPR-dCas9-gRNA-expressing EBV-positive cells in each cell line succumbed following ganciclovir treatment. Similar results were observed in EBV-positive gastric cancer Seoul National University (SNU)-719 cells through plasmid transfection for more transient CRISPRa delivery [66].

#### 3.2.4. CRISPR Systems Targeting Lytic Human Cytomegalovirus Replication and Reactivation

Genes encoding the HCMV IE proteins 1 and 2 were targeted using CRISPR-Cas9 in two studies [67,68]. Targeting the *UL122/123* gene with a singleplex (one gRNA) or multiplex (three gRNAs) strategy in MRC5 fibroblast cells, followed by HCMV infection, reduced IE1 and IE2 expression but failed to prevent late viral replication events due to unstable Cas9 expression in this cell line following lentiviral transduction [67]. Applying the multiplex strategy in a different cell line (HCMV-permissive astrocytoma cell line U-251 MG) did reduce the number of IE-positive cells by up to 95% and inhibited the genome replication cycle and late viral glycoprotein B production, therefore decreasing the virion release by at least 80% [67]. In the second study [68], IE1 and IE2 expression were targeted in human foreskin fibroblast cells through lentiviral delivery of a more stably expressed CRISPR-Cas9 system targeting a region upstream of the IE-coding gene. A decrease in viral protein production levels was observed, together with a reduction in viral DNA by 25-50% and virion production by 66%. CRISPR-Cas targeting of *IE* in monocytic THP-1 cells, followed by HCMV infection, reduced viral load and viral protein production upon induction of reactivation [68].

One study [45] observed impaired HCMV replication in MRC5 cells upon CRISPR-Cas9-mediated editing of different early HCMV genes, but not of nonessential HCMV genes, using lentiviral delivery of the CRISPR-Cas9 system.

Finally, three studies [45,69,70] used the CRISPR-Cas9 system to edit late HCMV genes. In the first study [45], lentiviral transduction of MRC5 cells with Cas9 and gRNAs against *UL86*, encoding the major capsid protein, followed by HCMV infection, impaired viral replication. The second and third study [69,70] used a different approach, namely viral interference, which implies co-infection of cells with a modified virus carrying Cas9 and gRNA against wild-type (WT) genes, called the gene drive virus, and a WT HCMV strain. Cleavage by Cas9 of a target sequence of the WT virus genome, followed by homologous recombination using the gene drive virus DNA as a template, leads to the conversion of the WT virus into new gene drive viruses, therefore driving the native viral population to extinction [69,70]. Gene drive viruses targeted against WT *UL23*, encoding an interferon-γ-inhibiting tegument protein, reached up to 95% of the final population and drastically suppressed WT viral infection [70]. Additionally, gene drive viruses against the tegument genes *UL26* and *UL35* decreased WT viral replication in fibroblasts [69].

#### 3.2.5. CRISPR Systems Targeting the Integrated Latent Human Herpesvirus 6A Genome

So far, only one study [37] employed the CRISPR-Cas9 system to remove the integrated HHV-6A genomes from the host telomeres of HHV-6A-infected cells. Prolonged Cas9 expression in HHV-6A-infected HEK293T cells upon lentiviral transduction of Cas9 together with plasmid transfection of multiple gRNAs targeting the noncoding region of the direct repeat regions (DRs) between the DR1 and the DR6/7 locus reduced the HHV-6A genome copies by about 70% compared to the control. Transient Cas9 expression through transfection with a CRISPR-Cas9-expressing plasmid decreased the HHV-6A genome in 65% of the cells, further decreasing in up to 80% of the cells when a second round of the transient plasmid delivery was performed 3 weeks later. The study also demonstrated the ability of the CRISPR-Cas9 system to excise the inherited chromosomally integrated HHV6A (iciHHV-6) genomes in approximately 57% of transfected smooth muscle cells (SMCs) [37].

#### 3.2.6. CRISPR Systems Targeting Kaposi’s Sarcoma-Associated Herpesvirus

##### The Application of CRISPR Systems to Inhibit Lytic KSHV Replication

Three studies [38,71,72] used the CRISPR-Cas system to edit lytic KSHV genomes. Targeting the IE gene *ORF57* in high copy number KSHV-infected body cavity-based lymphoma (BCBL)-1 cells using two gRNAs and Cas9 delivered via a single expression vector reduced both viral genome copy numbers and lytic gene expression [71]. Knockout of *ORF57* in human renal cell carcinoma inducible (i)SLK cells transfected with a KSHV bacterial artificial chromosome (BAC) clone (Bac16) did not result in viral genome instability, as the number of viral genome copies remained the same. However, upon lytic induction, the lytic viral replication was strongly attenuated as the *ORF57* gene knockout had a detrimental effect on virion production [71]. CRISPR-Cas9-mediated targeting of the immediate early gene *ORF45* induced INDELs and reduced KSHV DNA load in SLK cells [72]. Finally, CRISPRi has been used to silence the expression of *ORF57* or the delayed-early gene *ORF59*. Lentiviral delivery of dCas9-Krüppel-associated box (KRAB) and gRNA targeting the promoter of *ORF57* or *ORF59* in KSHV latently infected iSLK-219 cells, followed by induced reactivation, led to ~90% knockdown of the expression of the respective gene. Consequently, targeting both genes substantially reduced KSHV lytic L gene (*K8.1* gene) expression and viral titers [38].

##### The Application of CRISPR Systems Targeting KSHV Genes Important for Latency Maintenance

Several studies [38,44,72,73] applied CRISPR systems to target the expression of latency-associated nuclear antigen (LANA), a crucial KSHV latency protein encoded by *ORF73*. Transducing latently KSHV-infected Vero219 epithelial cells, L1T2 human endothelial cells, and human pleural effusion B lymphoblasts (BC3 cells) with an AAV5 encoding Cas9 and LANA-targeted gRNAs resulted in decreased LANA copy numbers and overall reduced KSHV episome burden [44]. The effects were time- and dose-dependent as they became more pronounced with time and increasing MOI. However, despite the loss of KSHV episomes, neither cell survival nor cell replication was affected [44]. In a different study [73], lentiviral delivery of the same CRISPR-Cas9 system in rat primary embryonic mesenchymal precursor cells (MM cells), which turn into tumor cells (KMM cells) following KSHV infection, resulted in no detectable expression of KSHV latency-associated genes, suggesting elimination of the viral episome. Importantly, KSHV episome elimination seemed to reverse the KSHV-mediated malignant transformation of the MM cells back to a normal state [73]. Transfection of BCBL-1 cells with liposome-encapsulated CRISPR-Cas9 ribonucleoprotein complexes targeting the LANA gene inhibited cell proliferation and induced cell apoptosis [73]. CRISPR-Cas9-mediated editing of the *ORF73* gene decreased the KSHV DNA quantity in KSHV-infected SLK cells [72]. Lastly, lentiviral transduction of latently KSHV-infected iSLK-219 cells and BCBL-1 cells with a CRISPRi system, consisting of dCas9-KRAB and gRNAs targeting the LANA promoter latent transcript (LT)c and/or LTi, substantially reduced LANA production in both cell lines and resulted in the loss of viral episomes in the transfected iSLK-219 cells [38].

##### The Application of CRISPR Systems to Induce the Reactivation of Latent KSHV Infection

Two studies [40,74] applied the CRISPR system to reactivate KSHV from its latent state into the lytic replication cycle. In a first study [40], activating the expression of the lytic IE *ORF50* gene with a CRISPRa system in KSHV-infected HEK293 cells resulted in increased viral genome copies, the expression of *ORF50* and other IE, E, and L genes, and infectious virion production, indicative of full KSHV lytic replication cycle initiation. This effect was even more pronounced when two sites were targeted simultaneously [40]. In the second study [74], the CRISPR-Cas9 system was applied in two latently KSHV-infected primary effusion lymphoma (PEL) cell lines to downregulate the expression of viral microRNAs (miRNAs), which resulted in slower cell growth and increased lytic gene expression without affecting latent gene expression. Interestingly, as viral miRNAs influence host gene expression and metabolism, corresponding alterations in both were also observed following CRISPR editing [74].

#### 3.2.7. Applying CRISPR Systems for the Enhanced Efficiency or Development of Cellular Immunotherapy

Patients receiving an allogeneic hematopoietic stem cell transplant (HSCT) are subjected to severe glucocorticoid treatment to avoid various complications related to graft-versus-host disease [75]. However, impairment of the host’s immune system by glucocorticoid treatment is frequently followed by reactivation of latent herpesvirus infections such as HCMV and EBV. In order to control life-threatening complications associated with virus reactivation, immunosuppressed patients can be infused with virus-specific T cells (VSTs). However, the survival of these cells is unstable as binding of the glucocorticoids to the glucocorticoid receptor (GR), encoded by *NR3C1* (*nuclear receptor subfamily 3 group C member 1*), of the VSTs leads to apoptosis and/or inhibition of proliferation [75]. Therefore, three studies [75,76,77] created HCMV- and/or EBV-specific *NR3C1*-knockout VSTs that are resistant to the lymphocytotoxic effect of glucocorticoids. This was achieved upon electroporating human peripheral blood mononuclear cells (PBMCs) with CRISPR-Cas9 ribonucleoproteins targeting exon 2 of *NR3C1*. The modified VSTs showed high viability and high proliferation capacity in vitro in the presence of dexamethasone without alteration of their maturation phenotype, effector function, cytotoxic activity, or cytokine release [75,76,77]. In vivo experiments infusing immunodeficient mouse models with the *NR3C1*-KO VSTs and dexamethasone showed a high frequency of human T cells in the bone marrow [75]. Eventually, good manufacturing practices (GMP)-grade *NR3C1*-KO VSTs were manufactured for clinical implementation [75,77].

Another study [43] manufactured EBV-specific cytotoxic T cells (CTLs) targeting EBV-associated gastric carcinoma cells (EBVaGC cells) by sensitization of peripheral blood lymphocytes with the EBV-LMP2A antigen. However, upregulation of programmed cell death protein 1 (PD-1) on the EBV-LMP2A-CTLs was observed along with the impairment of their cytotoxic function, leading to the hypothesis that EBVaGC cells could escape the CTL killing effect. Therefore, PD-1 was knocked out in EBV-LMP2A-CTLs using CRISPR-Cas9, leading to enhanced secretion of IFN-γ and in vitro cytotoxic activity against EBVaGC cells. This was confirmed in vivo, as human EBVaGC xenografted mice showed improved survival and higher cytokine release upon infusion of PD-1-disrupted LMP2A-CTLs compared to controls. Interestingly, the combination of low-dose radiotherapy and consequent immunotherapy of the modified CTLs led to significant tumor regression [43]. Additionally, one study [78] applied CRISPR-Cas9 editing in PBMCs to insert a green fluorescent protein (GFP)-Barcode transgene into EBV-specific T cells through HDR. Since the latter occurs primarily during active phases of the cell cycle, reactive T cells with enhanced EBV-specificity and cytotoxicity against EBV-lymphoblastoid cell lines in vitro can be selected [78].

Braun et al. [79] applied the CRISPR-Cas9 system to create CAR-T cells against EBV-infected Burkitt lymphomas by knocking out the *TRAC* (*T cell receptor-α constant*) gene and knocking in EBV glycoprotein 350 chimeric antigen receptor (gp350 CAR) or cluster of differentiation 19 (CD19) CAR templates via homology-directed repair. The CD19^KI^CAR-T cells showed strong cytotoxic effects against two human Burkitt lymphoma cell lines. Gp350^KI^CAR-T cells could efficiently kill HEK293T cells stably expressing EBV gp350, but exhibited only mild cytotoxic effects against the two human Burkitt lymphoma cell lines. Similarly, CD19^KI^CAR-T cells, but not gp350^KI^CAR-T cells, could significantly reduce the EBV DNA load in the bone marrow of mice xenografted with these Burkitt lymphoma cell lines. These observations could be explained by the weaker and more variable expression of gp350 instead of CD19 [79].

**Table 2:** Overview of the design from studies applying CRISPR-based antiviral therapy for herpesvirus infections.

Virus	Mechanism	Target	CRISPR System	Vector	Test Subject	Reference
HSV-1	Inhibiting lytic viral replication	Immediate early (IE) viral genes				
		**Singleplex**				
		*ICP0*	CRISPR-Cas9	Plasmid transfection	In vitro: Vero L7 cells	P. C. Roehm et al. [42]
				Lentivirus	In vitro: Human oligodendroglioma TC620 cells	
				Lentivirus	In vitro: Vero cells	D. S. Karpov et al. (2022) [41]
			CRISPR-Cas9, CRISPR-Cas9	Lentivirus	In vitro: Vero cells	Y. Chen et al. [52]
		*ICP4*	CRISPR-Cas9, CRISPR-Cas9	Lentivirus	In vitro: Vero cells	Y. Chen et al. [52]
			CRISPR-Cas9	AAV-1	In vitro: Murine primary trigeminal ganglion neuronal cells	
				Lentivirus	In vitro: Human foreskin fibroblasts	H. S. Oh et al. [56]
			ALICE_Cas9+gRNA_	AAVrh10	In vivo: Herpes simplex keratitis mouse model	Y. D. Wang et al. [59]
		*ICP27*	CRISPR-Cas9	Lentivirus	In vitro: Human foreskin fibroblasts	H. S. Oh et al. [56]
			CRISPR-Cas9	Lentivirus	In vitro: Vero cells	F. R. van Diemen et al. [45]
		**Multiplex**				
		*ICP0* (2 sites), *ICP27* (2 sites)	CRISPR-Cas9	Plasmid transfection	In vitro: Vero cells	N. Amrani et al. [50]
				AAV2	In vitro: Vero cells	N. Amrani et al. [50]
						A. Bellizzi et al. [51]
		*ICP0*-*ICP27*	CRISPR-Cas9	Lentivirus	In vitro: Human oligodendroglioma TC620 cells	P. C. Roehm et al. [42]
			CRISPR-Cas9	- AAV8-Y733 - AAV9	In vivo: HSV-1 latently infected rabbit keratitis model	N. Amrani et al. [50]
		*ICP0*-*ICP4*, *IPC4*-*ICP27*	CRISPR-Cas9	Lentivirus	In vitro: Human oligodendroglioma TC620 cells	P. C. Roehm et al. [42]
		Early (E) viral genes				
		**Singleplex**				
		*UL5*, *UL8*, *UL9*, *UL42*, or *UL52*	CRISPR-Cas9	Lentivirus	In vitro: Vero cells	F. R. van Diemen et al. [45]
		*UL19*	CRISPR-Cas9	Plasmid transfection	In vitro: Vero cells	D. S. Karpov et al. (2022) [41]
		*UL29*	CRISPR-Cas9	Lentivirus	In vitro: Human foreskin fibroblasts	H. S. Oh et al. [56]
			CRISPR-Cas9	Engineered extracellular vesicles	In vitro: - Vero cells - Hela cells - Neuro HT22 cells In vivo: Mouse model	Y. Wan et al. [58]
		*UL30*	CRISPR-Cas9	Lentivirus	In vitro: Vero cells	F. R. van Diemen et al. [45]
			CRISPR-Cas9	Lentivirus	In vitro: Human foreskin fibroblasts	H. S. Oh et al. [56]
			CRISPR-Cas9, CRISPR-CasX	Plasmid transfection	In vitro: Vero cells	D. S. Karpov et al. (2019 and 2022) [41,54]
		*UL39*	CRISPR-Cas9	Plasmid transfection	In vitro: Vero cells	J. Vasques Raposo et al. [57]
					In vivo: BALB/c mouse model	R. M. P. de Sousa [53]
		**Multiplex**				
		*UL8*-*UL29*	CRISPR-Cas9	Lentivirus	In vitro: - Vero cells - MRC5 human lung fibroblasts	F. R. van Diemen et al. [45]
			CRISPR-Cas9	Plasmid transfection	In vitro: Vero cells	D. S. Karpov et al. (2019 and 2022) [41,54]
				HELP (HSV-1-erasing lentiviral particle)	In vitro: - HEK293T cells - HaCaT cells - Murine primary corneal stromal cells In vivo: Herpes simplex keratitis mouse model Ex vivo: Human cornea	D. Yin et al. [49]
					Clinical trial in humans	A. Wei et al. [47]
		*UL29-UL52*	CRISPR-Cas9	Lentivirus	In vitro: - Vero cells - MRC5 human lung fibroblasts	F. R. van Diemen et al. [45]
			CRISPR-Cas9	Plasmid transfection	In vitro: Vero cells	D. S. Karpov et al. (2019 and 2022) [41,54]
		*UL8-UL52*	CRISPR-Cas9	Lentivirus	In vitro: Vero cells	F. R. van Diemen et al. [45]
		*UL19*-*UL30*	CRISPR-Cas9	Plasmid transfection	In vitro: Vero cells	D. S. Karpov et al. (2019 and 2022) [41,54]
		Late (L) viral genes				
		*gD*	CRISPR-Cas9	Plasmid transfection	In vitro: HEK293-AD cells	N. Khodadad et al. [55]
		*UL15*, *UL27*, *UL36*, or *UL37*	CRISPR-Cas9	Lentivirus	In vitro: Vero cells	F. R. van Diemen et al. [45]
		Nonessential viral genes/intergenic regions				
		*US3* or *US8*	CRISPR-Cas9	Lentivirus	In vitro: Vero cells	F. R. van Diemen et al. [45]
		Intergenic regions between *UL26-27* and *UL37-38*	CRISPR-Cas9	Lentivirus	In vitro: Human foreskin fibroblasts	H. S. Oh et al. [56]
		Host genes				
		*NECTIN-1*	CRISPR-Cas9	Lentivirus	In vitro: Human corneal epithelial cells	Y. Li et al. [62]
		*DUX4*	CRISPR-Cas9	Lentivirus	In vitro: - HEK 293T cells - HAP1 cells	E. Neugebauer et al. [61]
		Multiplex strategy (IE, E, L, and/or nonessential viral genes)				
		*ICP4*, *ICP27*, *VP16*, and/or *gD*	CRISPR-Cas9	Plasmid transfection Lentivirus	In vitro: BHK-21 cells In vivo: Mouse models	M. Ying et al. [60]
		*UL29*, *UL52*, and *US8*	ALICE_Cas9+gRNA_	Plasmid transfection	In vitro: HEK293 T cells In vivo: Mouse models	Y. D. Wang et al. [59]
	Targeting latent viral genomes for the inhibition of viral reactivation	**Singleplex**				
		*UL8* or *UL52*	CRISPR-Cas9	Lentivirus	In vitro: MRC5 human lung fibroblasts	F. R. van Diemen et al. [45]
		*UL29*	CRISPR-Cas9	Lentivirus	In vitro: MRC5 human lung fibroblasts	F. R. van Diemen et al. [45]
			CRISPR-Cas9	Lentivirus	In vitro: Human foreskin fibroblasts	H. S. Oh et al. [56]
		*UL30*	CRISPR-Cas9	Lentivirus	In vitro: Human foreskin fibroblasts	H. S. Oh et al. [56]
		**Multiplex**				
		*ICP0* (2 sites) or *ICP27* (2 sites)	CRISPR-Cas9	AAV2	In vitro: Human induced pluripotent stem cell-derived cerebral organoids	A. Bellizzi et al. [51]
		*UL29*-*UL30*	CRISPR-Cas9	Lentivirus	In vitro: Human foreskin fibroblasts	H. S. Oh et al. [56]
VZV	Inhibiting lytic viral replication and targeting latent viral genomes for the inhibition of viral reactivation	*ORF62/71*	CRISPR-Cas9	AAV2	In vitro: - Retinal-pigmented epithelial (ARPE-19) cells - Human embryonic stem cell (hESC)-derived neuron cultures	B. Wu et al. [48]
EBV	Targeting viral genes important for latency maintenance	**Singleplex**				
		*EBNA1* or *OriP*	CRISPR-Cas9	Plasmid transfection	In vitro: - NPC C666-1 cells - HEK293M81 cells	K. S. Yuen et al. [63]
				Lentivirus	In vitro: Burkitt’s lymphoma Akata-Bx1 cells	F. R. van Diemen et al. [45]
		W repeats	CRISPR-Cas9	Plasmid transfection	In vitro: - NPC C666-1 cells - HEK293M81 cells	K. S. Yuen et al. [63]
		*LMP1*	CRISPR-Cas9	Plasmid transfection	In vitro: Human NPC CNE-2 cells	H. Huo and G. Hu [64]
				PBAE-plasmid polyplex nanoparticles	In vitro: - Nasopharyngeal carcinoma C666-1 cells In vivo: - Mouse C666-1 xenograft tumor model	C. Yuan et al. [65]
					In vivo: KM mouse model	J. Ding et al. [39]
		*LMP2*	CRISPR-Cas9	Plasmid transfection	In vitro: Human NPC CNE-2 cells	H. Huo and G. Hu [64]
		**Multiplex**				
		*EBNA1*, *EBNA-LP*, EBNA-LP/PstI/125bp Repeats, *LMP1*, and *EBNA3C*	CRISPR-Cas9	Plasmid transfection	In vitro: Raji cells	J. Wang and S.R. Quake [46]
		*EBNA1* (2 sites) and *EBNA1*-*OriP*	CRISPR-Cas9	Lentivirus	In vitro: Burkitt’s lymphoma Akata-Bx1 cells	F. R. van Diemen et al. [45]
	Purposefully inducing viral reactivation	Promotor of *ZAT*	CRISPRa	Lentivirus Plasmid transfection	In vitro: - Akata (EBV^+^)/P3HR1Burkitt lymphoma cells - EBV^+^ gastric cancer SNU-719 cells - EBV^+^ nasopharyngeal carcinoma HK-1 cells	F.G. Sugiokto and R. Li [66]
	Enhancing the efficiency of cellular immunotherapy	EBV-VSTs				
		*NR3C1*	CRISPR-Cas9	Ribonucleoprotein electroporation	In vitro: Peripheral blood mononuclear cells	K. Koukoulias et al. [77]
		*CCR5*	CRISPR-Cas9	- Ribonucleoprotein nucleofection - AAV6	In vitro: Peripheral blood mononuclear cells	D. Palianina et al. [78]
		EBV-LMP2A-CTLs				
		PD-1	CRISPR-Cas9	Plasmid transfection	In vitro: Human peripheral blood lymphocytes In vivo: Mouse xenograft model	S. Su et al. [43]
	Developing cellular immunotherapy	CAR-T cells				
		*TRAC*	CRISPR-Cas9	Electroporation	In vitro: Peripheral blood mononuclear cells In vivo: Mouse xenograft models	T. Braun et al. [79]
HCMV	Inhibiting lytic viral replication and/or targeting latent viral genomes for the inhibition of viral reactivation	Immediate early (IE) viral genes				
		**Singleplex**				
		*UL122/123*	CRISPR-Cas9	Lentivirus	In vitro: MRC5 primary fibroblasts	J. Gergen et al. [67]
		*IE* (exon 1)	CRISPR-Cas9	Lentivirus	In vitro: - Human foreskin fibroblasts - THP-1 cells	J. Xiao et al. [68]
		**Multiplex**				
		*UL122/123*	CRISPR-Cas9	Lentivirus	In vitro: - MRC5 primary fibroblasts - U-251 MG astrocytoma cells	J. Gergen et al. [67]
		Early (E) viral genes				
		*UL44*, *UL54*, *UL57*, *UL70*, *UL84*, or *UL105*	CRISPR-Cas9	Lentivirus	In vitro: MRC5 human lung fibroblasts	F. R. van Diemen et al. [45]
		Late (L) viral genes				
		*UL86*	CRISPR-Cas9	Lentivirus	In vitro: MRC5 human lung fibroblasts	F. R. van Diemen et al. [45]
		*UL23*, *UL26*, or *UL35*	CRISPR-Cas9	Gene drive viruses	In vitro: Human foreskin fibroblasts	M. Walter et al. (2020, 2021) [69,70]
		Nonessential viral genes				
		*US6*, *US7*, or *US11*	CRISPR-Cas9	Lentivirus	In vitro: MRC5 human lung fibroblasts	F. R. van Diemen et al. [45]
	Enhancing the efficiency of cellular immunotherapy	HCMV-VSTs				
		*NR3C1*	CRISPR-Cas9	Ribonucleo-protein electroporation	In vitro: Peripheral blood mononuclear cells	T. Kaeuferle et al. [76]
						K. Koukoulias et al. [77]
					In vitro: - Peripheral blood mononuclear cells In vivo: - Mouse model	R. Basar et al. [75]
HHV-6A	Targeting the integrated latent virus	Noncoding region of DRs between the DR1 and the DR6/7 locus	CRISPR-Cas9	- Lentivirus - Plasmid transfection	In vitro: - HEK293T cells - Smooth muscle cells	G. Aimola et al. [37]
KSHV	Inhibiting lytic viral replication	*ORF54*	CRISPR-Cas9	Lentivirus	In vitro: Human renal cell carcinoma SLK cells	C. O. Haddad et al. [72]
		*ORF57*	CRISPR-Cas9	Puro vector	In vitro: BCBL-1 cells (body-cavity-based lymphoma cell line) iSLK/Bac16 cells	A. BeltCappellino et al. [71]
			CRISPRi	Lentivirus	In vitro: iSLK-219 cells	K. Brackett et al. [38]
		*ORF59*	CRISPRi	Lentivirus	In vitro: iSLK-219 cells	K. Brackett et al. [38]
	Targeting viral genes important for latency maintenance	*ORF73*	CRISPR-Cas9	AAV5	In vitro: - Vero219 cells - L1T2 human endothelial cells - BC3 human pleural effusion B lymphoblasts	F. Y. Tso et al. [44]
				- Lentivirus - Liposome	In vitro: - KSHV-transformed rat embryonic metanephric mesenchymal precursor cells (KMM cells) - BCBL-1 cells	E. Ju et al. [73]
				Lentivirus	In vitro: Human renal cell carcinoma SLK cells	C. O. Haddad et al. [72]
		LANA promoter LTc and/or LTi	CRISPRi	Lentivirus	In vitro: - iSLK-219 cells - BCBL-1 cells	K. Brackett et al. [38]
	Purposefully inducing viral reactivation	*ORF50* or *ORF57*	CRISPRa	Plasmid transfection	In vitro: HEK293 cells	E. Elbasani et al. [40]
		miR-K12-1 to -9 and miR-K12-11	CRISPR-Cas9	Lentivirus	In vitro: - BCBL-1 cells - BCP-1 cells	Z. P. Liang et al. [74]

## 4. Discussion

This review identified a broad range of CRISPR-based strategies that have been explored for targeting herpesvirus infections, highlighting their potential for future therapeutic applications. However, notable discrepancies in antiviral efficacy across studies targeting the same virus were observed. These inconsistencies may be attributed to differences in targeted genes, gRNA design, CRISPR systems, delivery vectors, host models, viral strains, methodologies for assessing antiviral activity, or experimental conditions. These different aspects will be discussed below with a focus on the balance between treatment efficiency and safety. Notably, only two of the eight human herpesviruses—HSV-2 and HHV-7—have not yet been targeted using CRISPR-based approaches, likely due to limited research focus or perceived lower clinical relevance.

### 4.1. Five Anti-Herpesviral Mechanisms of CRISPR Technologies

A total of five mechanisms were identified in the application of CRISPR technologies to interfere with herpesvirus infection. The first mechanism involves targeting viral genes (IE, E, and/or L genes) or host genes to abrogate the viral lytic replication cycle and thus the active phase of the infection. Although this mechanism leads to the successful reduction of virus production, it is the least interesting option. Antiviral drugs targeting lytic herpesvirus infection already exist, and the small time window for treatment during the active phase of the infection would complicate realistic clinical implementation. A more interesting approach includes the application of CRISPR systems in latently infected cells, which comprises three possible mechanisms. The first mechanism, targeting genes important for latency maintenance, is the most promising since it could permanently cure infection by removing viral genomes from host cells, which has been impossible with the existing antiviral drugs. Given that latent EBV and KSHV proteins are involved in the oncogenesis of EBV- and KSHV-associated malignancies, evaluating the effects of CRISPR systems on latent EBV and KSHV gene expression is particularly interesting. It is important to note that, despite the efficient reduction in the number of latent viral genomes by the CRISPR-Cas9 and CRISPRi systems, no study has achieved complete eradication of the latent EBV or KSHV episomes [38,39,44,45,46,63,64,65,72,73]. This is likely due to multiple factors such as the presence of multiple viral genome copies per cell, incomplete delivery of CRISPR components, chromatin-associated protection of episomes, and the potential survival advantage of cells retaining episomes. The second mechanism, inhibiting viral reactivation by targeting lytic genes in latent viral genomes, could help prevent a flare-up of clinical symptoms and reduce the risk of virus transmission. Importantly, only a small editing efficiency of HSV-1 latent viral genomes and no reduction in the number of latent viral genomes was observed. Consequently, the question arises whether CRISPR systems can directly target lytic genes in the latent genomes and prevent reactivation or only target lytic genes when reactivation is induced and subsequently inhibit lytic replication. The latter theory could be supported by the hypothesis that gRNAs would be unable to reach the targeted sequences due to heterochromatization of the viral genome during latency [9]. The third mechanism, purposefully inducing viral reactivation, can be used to sensitize the virus to the host immune system or antiviral drugs. This mechanism could be combined with the mechanism of targeting active lytic replication, thereby combining the antiviral effects of the host immune system, the current antiviral drugs, and CRISPR-Cas editing. Besides viral gene targets, host genes, such as *NECTIN-1* and *DUX4* for HSV-1, are an interesting choice as targeting the human genome instead of the viral genome could lessen the development of viral resistance. However, targeting sequences in the host genome poses biosafety risks as this could induce mutations in the human genome following NHEJ and must therefore be carefully evaluated prior to clinical implementation can be realized. A similar problem arises upon targeting the chromosomally integrated HHV-6A genomes as CRISPR-Cas9-mediated double-stranded cleavage in the host genome to excise the viral genome could induce pathological chromosomal rearrangements [80]. Similarly, host genes could be modified using CRISPR to generate EBV-specific cytotoxic T lymphocytes (CTLs) or modified virus-specific T cells (VSTs).

### 4.2. Four CRISPR Systems Are Used as an Anti-Herpesviral Strategy

Across all studies, a total of four CRISPR systems are employed, namely CRISPR-Cas9, CRISPR-CasX, CRISPR interference (CRISPRi), and CRISPR activation (CRISPRa), each differing in their structure and/or mode of action. Different mechanisms of the antiviral effect during lytic viral replication following the double-stranded DNA cleavage by the Cas9 endonuclease have been proposed: (1) disrupted viral genome packaging and consequent reduced virion production and transmission, (2) reduced production of viral proteins encoded by the targeted essential viral genes, and (3) induced genome instability, as a result of mutation induction following NHEJ, leading to the production of altered proteins with abnormal functionality [45]. The CRISPR-CasX system also implies RNA-guided double-stranded cleavage by an endonuclease CasX. However, the smaller size and higher specificity of CasX compared to Cas9 could be advantageous for vector packaging and clinical implementation [33]. Given that the CRISPRi and CRISPRa systems are based on transient transcriptional modulation without the induction of double-stranded breaks, these two CRISPR systems could be a safer option as they do not cleave the DNA, therefore circumventing the chance of mutation induction.

### 4.3. Delivery of CRISPR Systems

Multiple delivery vectors were used in the different studies, including viral vectors such as lentiviruses, AAVs, or gene drive viruses, and non-viral vectors such as plasmid transfection, RNP electroporation or nucleofection, nanoparticles, liposomes, and extracellular vesicles. Each delivery method has its pros and cons. First, the chosen vector should achieve efficient transfection or transduction of the CRISPR system intracellularly. In ex vivo and in vitro settings, non-viral methods such as DNA or RNP electroporation have demonstrated high efficiency and are frequently used, for example, in the optimization of VSTs (e.g., *NR3C1* knockout strategies [77]) or the generation of CAR T cells [79,81,82]. However, these techniques are not readily applicable to in vivo contexts, where tissue barriers complicate delivery to herpesvirus reservoirs like sensory ganglia and B lymphocytes. In such cases, viral vectors are often favored due to their superior transduction efficiency, capacity to traverse tissue barriers, and ability to sustain gene expression over time, as demonstrated in several in vivo studies [49,50,59,83,84,85]. Administering delivery vehicles locally (e.g., intrastromally in the eye) or decorating delivery vehicles with targeting ligands could be another strategy to enhance tissue penetration, while also increasing the specificity of CRISPR delivery and reducing off-target effects in healthy host cells. This could be achieved by adding specific binding ligands such as single-chain variable fragments (scFv) or nanobodies to delivery vehicles, a process typically easier in non-viral vectors, even though viral glycoproteins can also be modified with these ligands [86,87,88,89,90]. For instance, the targeting specificity of extracellular vesicles was significantly enhanced by fusion of a neuro-targeting RVG peptide with a membrane protein of the vesicle [58]. Notably, a vector specifically targeting herpesvirus-infected cells remains to be developed and could largely decrease toxicity toward healthy cells and thus biosafety concerns in general. This is largely due to a lack of biomarkers expressed on infected cells, especially latently infected cells [91]. Third, the packaging capacity of the vector should be large enough to package both the gRNAs and the Cas protein. The limited packaging capacity of the frequently used AAV vector could cause difficulties in delivering multiple gRNAs in a multiplex approach. Fourth, the duration of Cas expression intracellularly should be carefully considered to target enough viral genome copies for efficient antiviral activity while minimizing biosafety concerns. A more transient or HSV-1-specific Cas expression, as achieved with non-viral vectors, HELP particles, or the ALICE system, is more desirable than constitutive Cas expression to limit the chance of off-targets and the induction of host immune responses.

### 4.4. Treatment Window

CRISPR systems can be used either as a preventive or a treatment approach. Many studies are conducted in a preventive approach, transfecting or transducing the cells with the CRISPR system before herpesvirus infection. However, considering the ubiquitous spread and asymptomatic transmission of herpesvirus infection, delivering the CRISPR system post-herpesvirus infection in a treatment approach is clinically more interesting. Moreover, a preventive approach implies the persistent presence of the CRISPR system intracellularly, which entails prolonged Cas expression with associated biosafety risks. Additionally, a clinically reasonable time window for treatment should be established. In most studies, the antiviral effects only lasted a few days post-infection, suggesting a small time window for treatment. Given the high specificity of the CRISPR system, targeting sequences specific for a certain type of herpesvirus, the relevant system can only be administered after diagnosing the exact type of herpesvirus. Since a significant delay between the initial infection and the specific diagnosis is highly possible, the time window for treatment of acute lytic infection or reactivation should be realistically large enough for clinical implementation. However, this should again be weighed against the biosafety concerns associated with prolonged Cas expression. Alternatively, the virus could be targeted outside the acute phase, specifically the latent state, of which three approaches were described earlier.

### 4.5. Singleplex Versus Multiplex Strategy

CRISPR systems can be designed as a singleplex strategy, using only one gRNA to target a specific site, or as a multiplex strategy with multiple gRNAs targeting multiple sites within one gene or multiple genes simultaneously. Most studies [42,45,47,49,56,60,67] using both strategies observed a higher efficiency in reducing viral replication using the multiplex strategy compared to the singleplex strategy. This could be explained by the hypothesis that more sites are targeted and edited, resulting in more genome instability and subsequent disturbance of viral processes. Moreover, a multiplex strategy could have a higher chance of escaping resistance by the targeted virus, which implies the formation of mutations at the targeted cleavage site and subsequent circumvention of CRISPR-Cas9 editing [45]. More specifically, if one of the targeted sites manages to escape the binding of the gRNA by generating mutations in the target cleavage site, the remaining gRNAs can still direct Cas9 activity, ensuring continued, though potentially reduced, targeting efficiency of the viral genome. On the contrary, viral resistance development against the gRNA of the singleplex strategy could completely inactivate the antiviral activity of the CRISPR system. However, one disadvantage of the multiplex strategy is the higher risk for off-target editing since the higher number of gRNAs could target more sites with a similar sequence.

### 4.6. Barriers to Clinical Implementation

Achieving a balance between efficacy and safety remains a central challenge for the clinical application of CRISPR-based antiviral strategies. As discussed above, enhancing the activity or expression of CRISPR systems, particularly those that induce double-strand breaks, often increases the risk of off-target effects in healthy cells. In terms of total safety assessment of the CRISPR systems, three major factors should be taken into consideration: (1) off-target editing of the CRISPR system, (2) toxicity of the delivery vector of the system, and (3) activation of host immune responses against the CRISPR system or delivery vector. While not all studies performed safety assessment experiments, none of the ones that did [39,41,42,45,46,47,49,50,52,53,54,56,57,58,65,74,75,77] raised major concerns for the biosafety of the CRISPR systems. However, for biosafety assessment, careful consideration must be given to the choice of test subject used to evaluate the antiviral effects and safety of the CRISPR system, and whether these findings can be extrapolated to clinical applications in human patients. First, in vitro experiments conducted on non-human cell lines deliver the CRISPR system in cells containing a different host cell genome from the human genome. Consequently, when safety assessment experiments detect no significant off-target sites in the non-human genome, it is difficult to extrapolate this to human cells since the human genome could contain sequences complementary to the used gRNAs. Second, in vivo experiments conducted on mice or rabbit models could be unrepresentative of possible human host immune responses against the CRISPR system or the delivery vector. To date, no CRISPR-based therapy has been clinically approved for the treatment of herpesvirus infections, and only one phase I/II clinical trial has been performed (NCT04560790) [47] for the treatment of HSK. This highlights the major challenges in translating CRISPR-based anti-herpesviral approaches into clinical practice. Moreover, long-term safety data are currently lacking, underscoring the need for extended follow-up studies to fully evaluate the durability and potential risks of CRISPR-based anti-herpesviral therapies in human patients.

## 5. Limitations of the Study

Our scoping review aimed to systematically map the existing literature on CRISPR strategies that could be used as antiviral strategies against human herpesviruses. A key limitation of this review was the involvement of only two authors, which may have introduced a risk of bias during study selection, data extraction, and interpretation. Additionally, the inclusion of only English-language studies introduces the potential for language bias, as relevant publications in other languages may have been overlooked. Finally, the search strategy was limited to PubMed and Web of Science, potentially missing relevant studies found in other databases (e.g., Scopus), preprints (e.g., bioRxiv), or conference proceedings and other gray literature.

## 6. Conclusions

In conclusion, four different CRISPR technologies, CRISPR-Cas9, CRISPR-CasX, CRISPRi, and CRISPRa, have been applied so far for the potential treatment of HSV-1, VZV, EBV, HCMV, HHV-6A, or KSHV infection. The mechanisms of the treatment could be categorized into five categories, namely (1) the inhibition of lytic viral replication, (2) targeting viral genes important for latency maintenance, (3) targeting latent viral genomes for the inhibition of viral reactivation, (4) the purposeful induction of viral reactivation, or (5) enhanced efficiency or development of cellular immunotherapy. The efficient treatment of herpes simplex keratitis in patients without major biosafety concerns in the follow-up period shows the high potential of CRISPR technologies for antiviral therapy in clinical settings. However, the three main challenges for the clinical implementation of CRISPR technology include an efficient and specific delivery of the system inside the infected host cell, a reasonable time window for treatment with transient Cas expression, and the efficient prediction and monitoring of the biosafety of the CRISPR systems, especially long-term.

## Figures and Tables

**Table 1:** Overview of the searches performed in PubMed and Web of Science for each herpesvirus, with their associated number of search results.

Virus Type	Search	Number of Search Results
**PubMed**		
	CRISPR* AND (herpes* OR HHV)	422
	((“Clustered Regularly Interspaced Short Palindromic Repeats”[MeSH]) OR (“CRISPR-Cas Systems”[MeSH])) AND “Herpesviridae”[MeSH]	202
Herpes simplex virus type 1 and type 2/Human herpesvirus 1 and 2	CRISPR* AND HSV	115
	((“Clustered Regularly Interspaced Short Palindromic Repeats”[MeSH]) OR (“CRISPR-Cas Systems”[MeSH])) AND ((“Simplexvirus”[MeSH]) OR (“Herpesvirus 1, Human”[MeSH]) OR (“Herpesvirus 2, Human”[MeSH]))	61
Varicella-zoster virus/Human herpesvirus 3	CRISPR* AND (varicella-zoster virus OR VZV)	5
	((“Clustered Regularly Interspaced Short Palindromic Repeats”[MeSH]) OR (“CRISPR-Cas Systems”[MeSH])) AND “Herpesvirus 3, Human”[MeSH]	2
Epstein-Barr virus/Human herpesvirus 4	CRISPR* AND (Epstein-Barr virus OR EBV)	136
	((“Clustered Regularly Interspaced Short Palindromic Repeats”[MeSH]) OR (“CRISPR-Cas Systems”[MeSH])) AND “Herpesvirus 4, Human”[MeSH]	43
Human cytomegalovirus/Human herpesvirus 5	CRISPR* AND (cytomegalovirus OR CMV)	148
	((“Clustered Regularly Interspaced Short Palindromic Repeats”[MeSH]) OR (“CRISPR-Cas Systems”[MeSH])) AND “Cytomegalovirus”[MeSH]	27
Human herpesvirus 6	((“Clustered Regularly Interspaced Short Palindromic Repeats”[MeSH]) OR (“CRISPR-Cas Systems”[MeSH])) AND “Herpesvirus 6, Human”[MeSH]	0
Human herpesvirus 7	((“Clustered Regularly Interspaced Short Palindromic Repeats”[MeSH]) OR (“CRISPR-Cas Systems”[MeSH])) AND “Herpesvirus 7, Human”[MeSH]	0
Kaposi’s sarcoma-associated herpesvirus/Human herpesvirus 8	CRISPR* AND (Kaposi’s sarcoma-associated herpesvirus OR KSHV)	54
	((“Clustered Regularly Interspaced Short Palindromic Repeats”[MeSH]) OR (“CRISPR-Cas Systems”[MeSH])) AND “Herpesvirus 8, Human”[MeSH]	19
**Web of Science**		
	CRISPR* (All Fields) and herpes* (All Fields)	316
	CRISPR* (All Fields) and HHV* (All Fields)	53
Herpes simplex virus type 1 and type 2/Human herpesvirus 1 and 2	CRISPR* (All Fields) and HSV* (All Fields)	174
Varicella-zoster virus/Human herpesvirus 3	CRISPR* (All Fields) and varicella-zoster virus (All Fields)	6
	CRISPR* (All Fields) and VZV (All Fields)	5
Epstein-Barr virus/Human herpesvirus 4	CRISPR* (All Fields) and Epstein-Barr virus (All Fields)	166
	CRISPR* (All Fields) and EBV (All Fields)	98
Human cytomegalovirus/Human herpesvirus 5	CRISPR* (All Fields) and cytomegalovirus (All Fields)	103
	CRISPR* (All Fields) and CMV (All Fields)	98
Human herpesvirus 6	Included in the general search	0
Human herpesvirus 7	Included in the general search	0
Kaposi’s sarcoma-associated herpesvirus/Human herpesvirus 8	CRISPR* (All Fields) and Kaposi’s sarcoma-associated herpesvirus (All Fields)	44
	CRISPR* (All Fields) and KSHV (All Fields)	44

## Data Availability

All data are available in the manuscript.

## Abbreviations

AAV	Adeno-associated virus
ALICE	Autonomous, intelligent, virus-inducible immune-like
ARPE-19	Adult retinal pigment epithelial cells clone 19
BAC	Bacterial artificial chromosome
BALB/c	Bagg albino laboratory-bred substrain c
BCBL	Body cavity-based lymphoma
BHK	Baby hamster kidney
Cas	CRISPR-associated protein
CAR	Chimeric antigen receptor
CD	Cluster of differentiation
CLEAR	Coordinated lifecycle elimination against viral replication
CNE-2	Cantonese nasopharyngeal epithelial cells
CRISPR	Clustered regularly interspaced short palindromic repeats
CRISPRa	CRISPR activation
CRISPRi	CRISPR interference
CTLs	Cytotoxic T cells
dCas9	Deactivated Cas9
DR	Direct repeat region
dsDNA	Double-stranded DNA
DUX4	Double homeobox 4
E	Early
EBNA	EBV nuclear antigen
EBV	Epstein-Barr virus
EBVaGC	EBV-associated gastric carcinoma cells
EVs	Extracellular vesicles
gD	glycoprotein D
GFP	Green fluorescent protein
GMP	Good manufacturing practice
gp	Glycoprotein
GR	Glucocorticoid receptor
gRNA	guide RNA
HaCaT cells	Spontaneously immortalized human keratinocyte cells
HAP1	Haploid human cell line 1
HCMV	Human cytomegalovirus
HDR	Homology-directed repair
HEK293	Human embryonic kidney cells
HEK293T	Human embryonic kidney cells with SV40 large T antigen
HELP	HSV-1-erasing lentiviral particles
hESC	Human embryonic stem cell
HHV	Human herpesvirus
HSCT	Hematopoietic stem cell transplant
HSK	Herpes simplex keratitis
HSV	Herpes simplex virus
HT22	Hippocampal terminal cells
iciHHV-6	Inherited chromosomally integrated human herpesvirus 6
ICP	Infected cell protein
IE	Immediate early
IgG	Immunoglobulin G
INDELs	Insertions or deletions
(i)SLK	(Inducible) cell line from a Kaposi’s sarcoma lesion
KO	Knock-out
KRAB	Krüppel-associated box
KSHV	Kaposi’s sarcoma-associated herpesvirus
L	Late
LANA	Latency-associated nuclear antigen
LAT	Latency-associated transcript
LMP	Latent membrane protein
LP	Leader protein
LT	Latent transcript
MeSH	Medical subject heading
miRNA	microRNA
MOI	Multiplicity of infection
MRC5	Medical Research Council strain 5
mRNA	Messenger RNA
NECTIN-1	Nectin cell adhesion molecule 1
NHEJ	Non-homologous end joining
NPC	Nasopharyngeal carcinoma
NPs	Nanoparticles
NR3C1	Nuclear receptor subfamily 3 group C member 1
ORF	Open reading frame
OriP	Latent origin of replication
OSF	Open Science Framework
PBAE	Poly(β-amino ester)
PBMCs	Peripheral blood mononuclear cells
PD-1	Programmed cell death protein 1
PEL	Primary effusion lymphoma
PML	Promyelocytic leukemia
PRISMA-ScR	Preferred Reporting Items for Systematic Reviews and Meta-Analyses extension for Scoping Reviews
RVG	Rabies virus glycoprotein
ScFv	Single-chain variable fragment
SMC	Smooth muscle cells
SNU-719	Seoul National University cell line 719
STING	Stimulator of IFN genes
TC620	Human oligodendroglioma Tübingen cells
THP-1	Human monocytic leukemia cell line
TRAC	T cell receptor-α constant
UL	Unique long region
US	Unique short region
VP16	Viral protein 16
VSTs	Virus-specific T cells
VZV	Varicella-zoster virus
WT	Wild-type
ZTA	Z trans-activator
